# Meek Micro-Skin Grafting and Acellular Dermal Matrix in Pediatric Patients: A Novel Approach to Massive Extravasation Injury

**DOI:** 10.3390/jcm12144587

**Published:** 2023-07-10

**Authors:** Michele Maruccia, Pasquale Tedeschi, Claudia Corrao, Rossella Elia, Simone La Padula, Pietro G. Di Summa, Giulio M. M. Maggio, Giuseppe Giudice

**Affiliations:** 1 Division of Plastic and Reconstructive Surgery, Department of Precision and Regenerative Medicine and Ionian Area (DiMePRe-J), University of Bari Aldo Moro, Piazza Giulio Cesare, 70121 Bari, Italy; 2 Department of Plastic and Reconstructive Surgery, Università degli Studi di Napoli Federico II, Via Pansini 5, 80131 Napoli, Italy; 3 Department of Plastic and Hand Surgery, Centre Hospitalier Universitaire Vaudois (CHUV), University of Lausanne (UNIL), 1015 Lausanne, Switzerland

**Keywords:** extravasation injury, pediatric patients, Meek micrografting, dermal substitute, scar management

## Abstract

(1) Background: Extravasation injuries in pediatric patients can lead to significant harm if they are not promptly diagnosed and treated. However, evidence-based standardization on extravasation management remains limited, particularly for extensive wound necrosis. This case report presents the management of an 8-week-old premature patient with an extensive extravasation injury involving the right forearm and dorsum of the hand. (2) Methods: The patient was evaluated by a multidisciplinary team in our Neonatal Intensive Care Unit. Surgical intervention involved the debridement of necrotic tissues, followed by temporary coverage with an acellular dermal matrix. Definitive coverage was achieved through Meek micrografting after three weeks. Physical therapy was provided with pre- and post-rehabilitation range of motion assessed using goniometric measurements. Scar quality was evaluated using the Vancouver Scar Scale. (3) Results: The engraftment rate of the Meek micrografts was 93%, with 16 out of 226 micrografts lost. The patient achieved a Vancouver Scar Scale score of 6, indicating a moderate degree of scarring. Significant improvements in elbow, wrist, and finger joint range of motion were observed at a 1-year follow-up. (4) Conclusions: Close observation and heightened awareness of extravasation risks by trained personnel are crucial. Meek micrografting combined with dermal substitute coverage represents an innovative approach to managing extravasation wounds in pediatric patients.

## 1. Introduction

Extravasation injuries are a serious iatrogenic consequence of intravenous fluid administration, which can lead to soft tissue necrosis and skin damage resembling burns, especially when they affect the hand and forearm [1]. Although this kind of injury is uncommon, it happens frequently in children, with rates ranging from 11% to 58% after routine infusions and up to 38% leading to skin necrosis [2,3]. Due to the fragility and size of their veins as well as their immature skin, premature infants are more susceptible to these injuries [4]. Moreover, excessive osmotic imbalance caused by the administration of hypertonic solutions, such as total parenteral nutrition (TPN), can lead to tissue injury [5].

The severity of the tissue damage resulting from extravasation depends on various factors, such as the type of drug, the amount extravasated, and the location of the injury. Skin necrosis, infections, nerve or tendon damage, and compartment syndrome are among the potential complications that can result from extravasation [6]. These complications can cause long-term scarring, contractures, functional loss, and joint deformities, making it critical to take preventive measures and to identify and manage extravasation injuries promptly [7].

In such a scenario, pediatric patients’ healing is compromised by the limited donor sites for skin autografts required to achieve permanent wound coverage. Further, it is essential to minimize donor site scarring, reduce primary wound healing time, and achieve the best functional outcome [8]. Wound coverage using a standard skin graft, even if meshed, requires the presence of approximately an equal surface area at the donor site. Furthermore, not all areas of the skin surface can be used for harvesting a skin graft, either for functional reasons or for aesthetic ones [9,10].

The modified Meek technique of micrografting offers an alternative method of coverage for extensive areas in the absence of adequate donor sites [11]. This kind of procedure has always been considered a “rescue” technique for patients with major burns in which definitive coverage of the wounds could not be obtained with standard methods [12]. The Meek technique has several advantages, as it allows the skin graft to expand from 3 to 9 times and to predict the expansion ratio. It also allows one to obtain the maximum re-epithelialization edge thanks to islands of dermis and epidermis that are geometrically equal and equally spaced. It reduces the surface area to be harvested and consequently reduces complication rates [13,14]. Therefore, for pediatric patients suffering from extensive extravasation necrosis, the use of the modified Meek procedure could represent a cutting-edge technique for their healing.

This article presents the case of an 8-week-old patient who suffered massive necrosis of the entire right forearm and dorsum of the hand due to parenteral extravasation and received early multidisciplinary treatment, including Meek micrografts.

## 2. Case Report

An 8-week-old premature female patient was transferred to our Neonatal Intensive Care Unit (NICU) after experiencing an extensive extravasation injury that involved the entire right forearm and dorsum of the hand. The patient’s weight at the time of admission was 2.800 g, and her height was 46 cm.

The etiological agent underlying the patient’s medical condition was traced back to total parenteral nutrition (TPN), which was administered via intravenous access in the right forearm for maintenance purposes. The patient had received TPN as part of her treatment for an inflamed Meckel diverticulum, a rare condition characterized by the protrusion of a small pouch in the intestinal wall, and before that, she had had no relevant past interventions.

**Figure 1:**
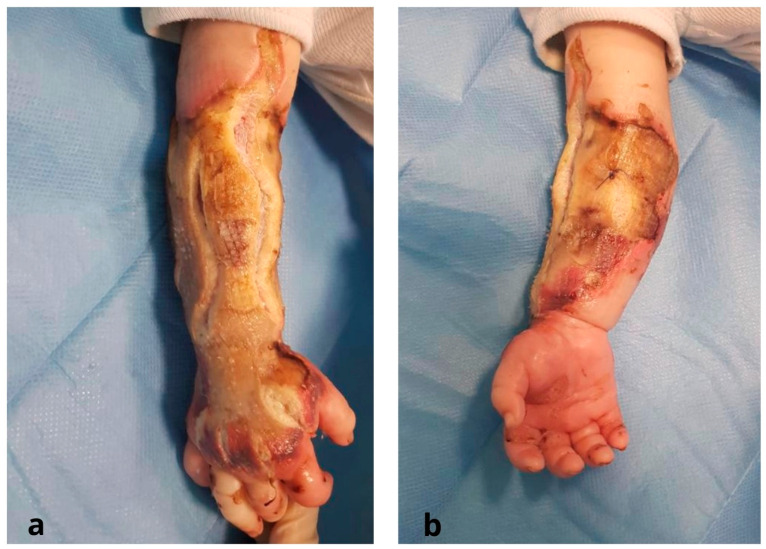
Pre-operative stage: (**a**) dorsal region of the right forearm and hand after fasciotomies; (**b**) volar region of the right forearm.

Prior to the transfer, fasciotomies of both the dorsum of the hand and forearm had been carried out on the upper limb (Figure 1).

After being transferred to the NICU of our institution, the patient was evaluated by a multidisciplinary team. The patient was found to have an increased level of procalcitonin (67.3 ng/mL), PCR (62 mg/L), D-Dimer (6750 µg/L), WBC (23.7 × 10^3^/uL), and an increased body temperature (38.7 °C). Pathogenic microorganisms were valued directly from clinical specimens: serial blood and urinary culture tests were performed, along with rectal, pharyngeal, and auricular swabs. Urinary and blood cultures tested negative, as did the rectal swab, whereas the pharyngeal and auricular swabs tested positive for Staphylococcus hominis. Thus, treatment with Amoxicillin and clavulanic acid was started.

**Figure 2:**
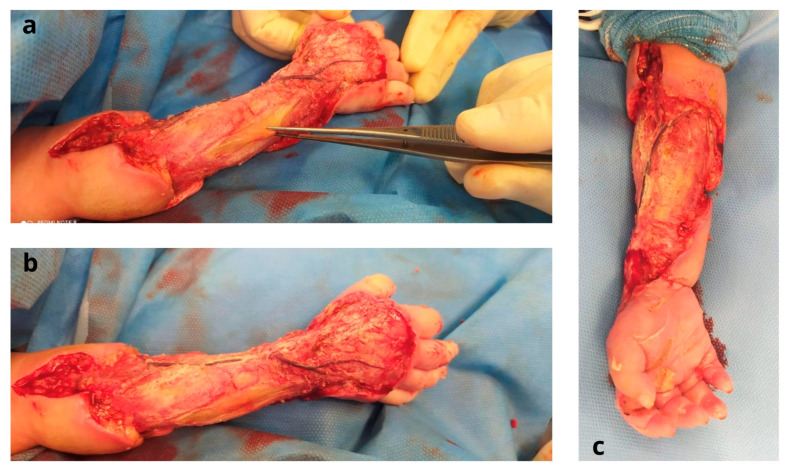
Intra-operative stage after surgical debridement of skin and necrotic tissues: (**a**,**b**) dorsal region of the forearm with exposure of common extensor muscles; (**c**) volar region of the forearm.

**Figure 3:**
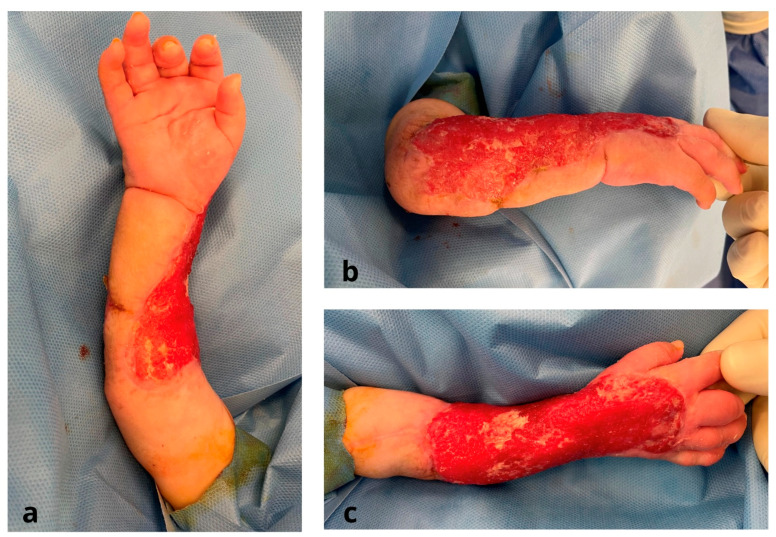
Intra-operative stage after removal of Pelnac: (**a**) volar regione of the forearm; (**b**,**c**) dorsal region of the forearm.

Two days following her admission to the NICU, a surgical intervention under general anesthesia was carried out (Figure 2). The common extensor muscle of the fingers had critical ischemia, and the superficial venous system was dilated and thrombosed. Various cultured biopsies of the wound were taken and sent for further analysis. A surgical debridement involving skin and necrotic soft tissues was performed, and temporary coverage of the area was accomplished with an acellular dermal matrix (Pelnac^®^, Gunze Corp., Osaka, Japan) (Figure 3).

Culture biopsies tested positive for the Enterobacter cloacae complex; therefore, the initial antibiotic regimen was switched to Meropenem. Several dressings were applied using vaseline gauze and sterile gauze soaked in chlorhexidine.

The modified Meek technique for split-thickness skin graft (STSG) expansion was taken into account by the multidisciplinary team, as micrografting would allow the coverage of large areas while minimizing the amount of surface tissue required from donor areas.

A list of anatomical areas to be grafted, in order of priority, was developed. Areas where successful grafting would be expected, such as the lower limbs, were addressed first. Areas such as the back, where continuous pressure and possible friction threatened graft viability, were excluded.

**Figure 4:**
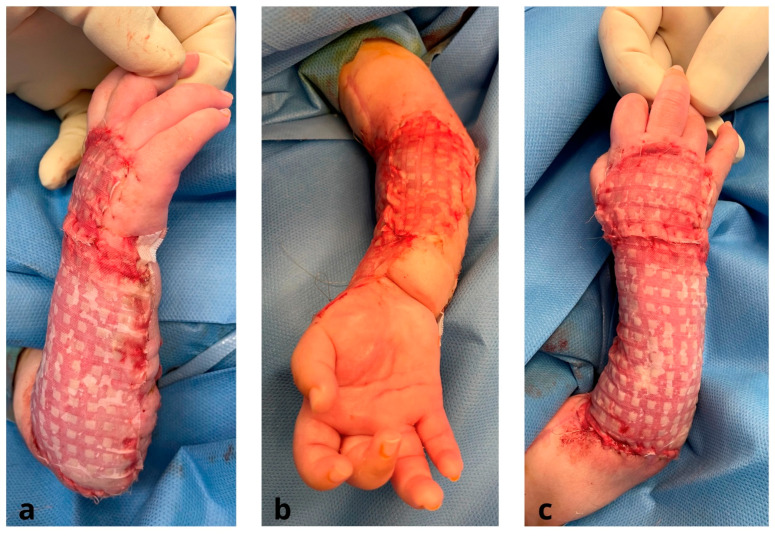
Intra-operative stage after micrograft implantation using modified Meek Technique: (**a**,**c**) dorsal region of the forearm; (**b**) volar region of the forearm.

**Figure 5:**
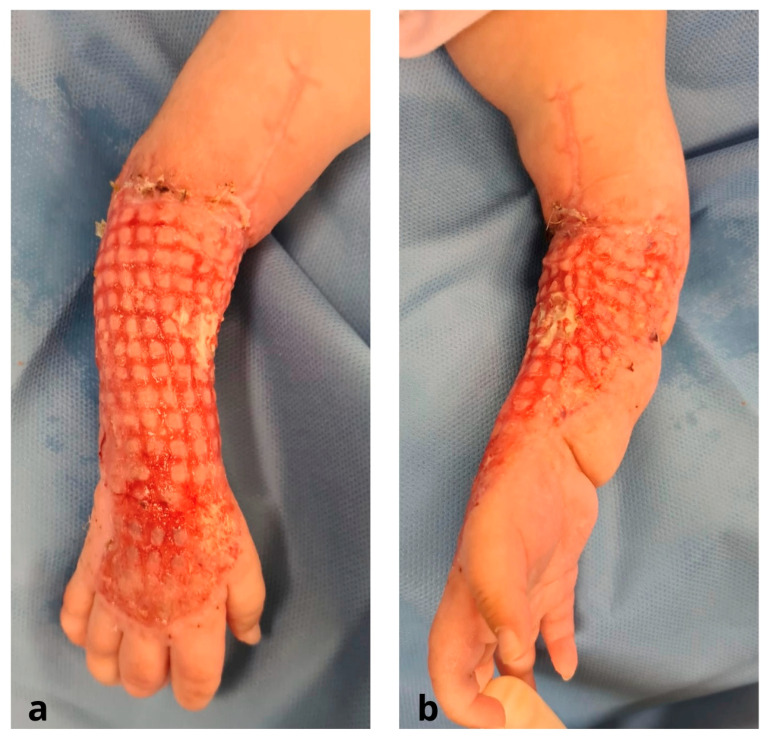
Intra-operative stage after removal of Meek scaffolds: (**a**) dorsal region of the forearm; (**b**) volar region of the forearm.

The grafts were placed dermal side down onto square cork supports. The meshing device was then equipped with cork supports, and 13 blades split the graft into 196 islands of skin graft measuring 3 mm by 3 mm each. This micrograft material was applied to a piece of folded polyamide gauze. By applying traction to the gauze’s edges, it opened up. Once over the wound bed, the unfolded gauzes were secured with 4/0 monocryl (Figure 4). After 6–7 days, the Meek micrografts were sufficiently stable and adhered to the wound bed, enabling the removal of the scaffold. The micrografts were covered with Vaseline dressings, which were left in place for an additional week. After micrograft implantation, each Meek graft underwent re-epithelialization from its edges, covering the vast majority of the wound bed. Two to three weeks postoperatively, there was a gradual attenuation of the Meek micrografts, giving rise to a more discreet appearance that was esthetically superior to that of traditionally meshed STSGs (Figure 5).

**Figure 6:**
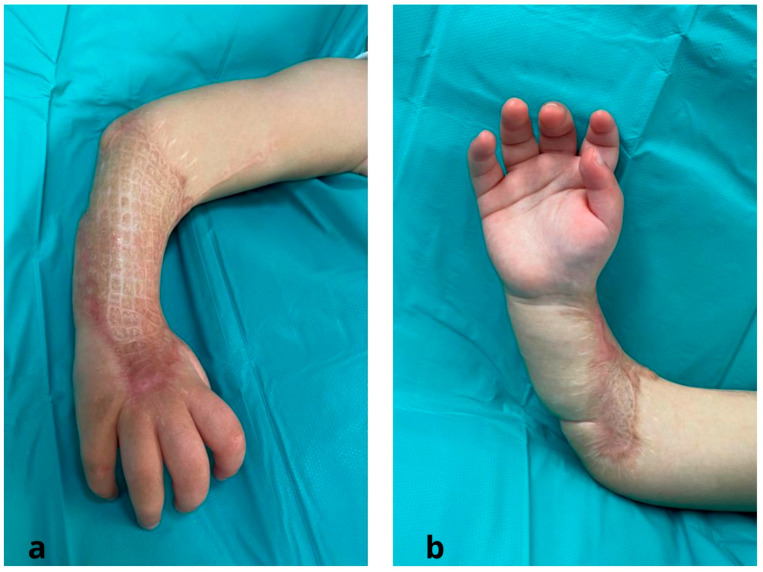
Micrografts at one-year follow up after surgery: (**a**) dorsal region of the forearm; (**b**) volar region of the forearm.

The Meek technique was used just once to completely cover the injured areas. The only donor site was the right thigh. After hospital discharge, the patient underwent a comprehensive and tailored physical therapy program to optimize postoperative outcomes. The patient was followed in an outpatient setting at weekly intervals for 1 month and at 6 and 12 months. At the latest follow-up (12 months), no pathological evolution of the scars was observed; z-plasties are planned to follow the lengthening of the limb (Figure 6).

The engraftment rate of the Meek micrografts was assessed. Functional deficits in the range of movement of the elbow, wrist, and finger joints were evaluated using goniometric measurements. Furthermore, the quality of the scars achieved was evaluated using the Vancouver Scar Scale (VSS).

Our report follows the Consensus-based Clinical Case Reporting (CARE) Guideline Development, validated by the Enhancing the QUAlity and Transparency Of health Research (EQUATOR) network [15].

## 3. Results

Out of a total of 226 micrografts transplanted, 210 survived and 16 were lost, resulting in an engraftment rate of 93%. The VSS score for this patient was 6, indicating a moderate degree of scarring. While the patient did not achieve a perfect score, the scars were still considered acceptable and represented a significant improvement from the initial injury.

The patient had an impaired elbow joint and was unable to extend the first finger. Following surgical intervention, a comprehensive and tailored physical therapy program incorporating both passive and active range-of-motion exercises was implemented to optimize postoperative outcomes.

Follow-up evaluations conducted at 6 months and 1 year post-surgery demonstrated a remarkable improvement in the range of motion of the elbow and wrist. Notably, goniometric measurements of the elbow joint revealed an increase in passive flexion from 130 degrees to 140 degrees, signifying a notable gain in motion. Conversely, passive elbow joint extension improved from 50 degrees to 80 degrees.

The wrist joint showed a neutral position of extension of 10 degrees due to scar retraction on the dorsal surface of the hand. The goniometric measurements of the wrist joint showed an improvement in passive flexion from 25 degrees to 60 degrees. Additionally, passive wrist joint extension improved from 30 degrees to 50 degrees. Unfortunately, the range of motion of the first finger did not improve because of the severe damage to the extensor tendon (Table 1).

## 4. Discussion

The creation of novel, wound-management-friendly biomaterials is the result of advancements in tissue bioengineering. Dermal substitutes are biomaterials that offer a temporary or permanent replacement for damaged skin tissue. They provide a neodermis that can be used as a substrate for the later placement of a skin graft or as a standalone replacement for damaged skin. Dermal substitutes can be made of natural or synthetic materials and can be tailored to meet specific requirements, such as promoting wound healing, reducing scarring, and minimizing the risk of infection. The use of dermal substitutes has numerous advantages over traditional skin grafts, such as reducing the need for large donor sites, improving wound bed preparation, and providing a more natural and esthetically pleasing result. Additionally, dermal substitutes have shown promise in promoting the regeneration of dermal tissue, reducing scarring, and restoring skin function.

In the field of reconstructive surgery, advancements in the utilization of acellular dermal matrices (ADM) have opened up new possibilities for the management of large skin defects. One such approach involves the co-grafting of ADM and split-thickness skin grafts (STSG), which has shown promising outcomes in various applications.

In 2022, Gierek et al. reported favorable results in the treatment of hidradenitis suppurativa (HS), a chronic inflammatory disease. The authors reported an efficient wound healing process with flexible scars and an improved quality of life for the patients. To evaluate the healing process, the authors employed novel methodologies such as speckle laser analysis (LASCA), reporting an increased hyperemia, indicating healing and vascularization. Assessments using Cutometer^®^ dual MPA 580 showed positive outcomes, and skin ultrasound confirmed the thickness of ADM and STSG. Flexible scars and improved quality of life were observed [16]. Overall, the development and use of dermal substitutes have significantly improved wound management and have the potential to revolutionize the field of tissue engineering [17].

Flaps are a common method of reconstructing soft tissue defects, but they may not always be suitable for pediatric patients. In the case of an 8-week-old premature patient, the use of a flap to reconstruct the forearm would not have been the best option for several reasons. First, the patient’s skin and soft tissue are still developing and may not have the capacity to withstand the trauma of flap surgery. Second, the size of the defect in this case was extensive and involved a significant amount of tissue loss, making it difficult to harvest enough tissue for a flap [18]. Additionally, flap surgery carries a risk of complications such as flap necrosis, hematoma, and infection, which can be especially detrimental in young patients.

Therefore, the decision to use temporary coverage of the wound with a Pelnac^®^ dermal substitute and to follow up after 3 weeks with a split-thickness skin graft (STSG) was made in order to minimize the risks associated with flap surgery and to achieve the best possible outcome for the patient.

Being an 8-week-old pediatric patient, the harvesting site of STSG was extremely critical, having to minimize the need for a re-operation and lower donor site morbidity.

Although donor site wounds are created under optimal circumstances, they sometimes cause a substantial burden to patients throughout and following the healing progression. They can be painful, become infected, cause itching, and have a poor cosmetic appearance. Thus, always keeping the donor site minimal is advisable [19].

To overcome this clinical obstacle, several expansion techniques have been established over the years, some of which date back to the 1950s, but their use on children has always been limited.

Graft expansion provides a higher engraftment rate as it allows the drainage of any fluid or hematomas. It also shortens the patient’s hospital stay by allowing for faster wound healing [20].

Currently, the most popular method for skin autograft expansion is the meshing technique [21]. However, when this procedure is employed for graft expansion rates greater than 1:4, it produces skin grafts that are incredibly fragile and difficult to handle, making the technique less than ideal [22]. Minor expansions on mesh skin grafts (ratio 1:2 or less) may produce better cosmetic results; nevertheless, they have limited potential in terms of open wound coverage and donor site morbidity. Several authors have shown that performing a larger expansion leaves significant areas between the mesh interstices where the wound is exposed. These gaps can cause delays or even failures in re-epithelialization and higher rates of infection [23].

Conversely, the modified Meek micrograft technique allows for graft expansion, which relies on pre-folded scaffolds rather than skin graft stretching. This key element allows the skin graft to be spread smoothly, systematically, and in the right orientation. The application of a modified nylon scaffold and a special glue used in the Meek technique facilitates the surgical procedure and increases graft stability on the wound bed, reducing the risk of graft shifting and displacement [23].

In a recent 2022 article published by Noureldin et al., the authors compared the Meek technique with meshed grafts. In the study, the authors report that micrograft islands have a higher resistance to infection than traditional mesh grafts (25% vs. 40%) [24].

They also reported that, subjectively, the POSAS scar assessment scale had better results for the Meek group than for the mesh group (3.17 vs. 4.2; *p* = 0.048). The overall observer score was also better for the Meek group, with a mean general opinion score of 2.89, and for the mesh group it was 4.1 (*p* = 0.003). As a result, this technique allows for a proper and reliable surgical approach [24].

In the field of wound management, the quest for effective biological dressings as substitutes for human skin has been an ongoing and challenging endeavor. Severe traumas often lead to infections and a substantial loss of body fluids, including water, proteins, and electrolytes. Biological dressings have emerged as valuable substitutes for human skin in the management of wounds. Notably, allogeneic and xenogeneic dressings have garnered considerable attention due to their immediate availability for application, unlike cultured keratinocytes, which require a lengthy culture period of approximately 21 days. In our study, we aim to highlight the advancements achieved through the utilization of acellular dermal matrices (ADM) as a promising biological dressing in combination with Meek micrografting. Unlike conventional biological dressings, ADM provides a collagen-rich scaffold devoid of cells that can be rejuvenated with autologous cells to stimulate natural mechanisms of regeneration and reconstruction. The low immunogenicity of ADM further enhances its suitability as a biological dressing, making it an attractive option for wound management [25].

Some authors have proposed the application of cadaver skin as a biologic dressing to cover Meek’s micrografts; however, we decided to avoid such surgical procedures because these allogeneic materials have limited benefits when it comes to protecting Meek’s skin islands from infection [26]. In contrast, the re-epithelialization of the edges of the autografted Meek islets may be slowed down by the strong adhesion of overlapping allografts to the wound bed. Additionally, there is a potential concern that overlapping allografts may trigger donor-specific human leukocyte antigen sensitization [27].

The use of keratinocyte epithelial cultures is an alternative to the Meek technique. Nevertheless, the success rate of these cultures is erratic and unpredictable, and it has a much higher economic cost than Meek’s expansion [23].

In 2013, Menon et al. described the use of Meek micrografts in combination with keratinocyte cultures in a retrospective review of seven pediatric patients. This study brought to light how the use of both techniques determines an enhanced epithelialization process compared to the use of Meek alone [23].

In our case, we decided to avoid the use of keratinocyte cultures as we had already used a dermis substitute. This reconstructive option, as reported by Nicoletti et al. in 2019, represents a low-invasive procedure for fragile patients, such as children. Compared to keratinocyte cultures, such an approach can be cost-effective by increasing the chances of Meek islet engraftment. The need for a second batch of Meek Micrografts is rare [28].

To the best of our knowledge, no case of pediatric extravasation injury treated with the Meek technique has yet been reported in the literature.

Considering the advantages that the technique could bring in our specific case, we chose to apply this technique to an 8-week-old patient, obtaining an appropriate graft and stable wound coverage. In this case, we have achieved an engraftment rate of over 93%. The role of this association between the Pelnac^®^ dermal matrix and the Meek technique requires further investigation within highly specialized clinical settings.

Regarding functional and esthetic results, being a single case report, it is difficult to come to a definitive conclusion or make a valid comparison with mesh grafting techniques in patients of this age. However, we found that the appearance of the skin pattern, local pigmentation, and tissue pliability were good to excellent depending on the patient’s age, skin type, rate of expansion, and location of the donor site.

Clinical outcomes can vary significantly from patient to patient and depend on several contributing factors, such as genetic background, depth and extent of lesion, use of a dermal substitute, timing of intervention, and the presence of local infection or maceration [29].

## 5. Conclusions

The combined use of Pelnac^®^ as a dermal substitute with Meek STSG expansion for coverage of extensive upper limb extravasation wounds in pediatric patients is a reliable procedure.

The Meek micrograft method appears to be the best option for children for whom limiting donor site extension may be a key driver for better long-term outcomes.

Advantages over traditional meshed grafts in pediatric patients include the possibility of coverage of larger surface areas with limited donor site morbidity, a high engraftment rate, faster epithelialization, and similar or improved functional and esthetic results. The Meek technique works best on dermal substitutes, which can be applied over contaminated wound beds. The need for a second batch of Meek micrografts is infrequent.

## Figures and Tables

**Table 1:** Summary of patient timeframes, treatment details, and range of motion measurements.

Diagnosis	Age (Weeks)	Tretment	Range of Motion
Inflamed Meckel diverticulum	8	Total parenteral nutrition (TPN) via intravenous access in the right forearm for maintenance purposes (8 weeks)	
Extensive Extravasation Injury of soft tissues and skin of the right forearm	8	Fasciotomies of both dorsum of the hand and forearm	
	8	Multidisciplinary evaluation in Neonatal Intensive Care Unit (NICU)	
	8	Increased level of procalcitonin, PCR, D-Dimer, WBC, and body temperature. blood and urinary culture tests, rectal, pharyngeal, and auricular swabs for pathogen identification. Treatment with Amoxicillin and clavulanic acid (8–9 weeks)	
	9	Surgical debridement and temporary coverage with acellular dermal matrix (Pelnac^®^)	
Extensive Extravasation Injury with Exposed Tendons and Underlying Tissues	9	Culture biopsies confirming enterobacter cloacae complex infection, leading to antibiotic regimen switch to Meropenem	
	9–12	Dressings applied using vaseline gauzes and sterile gauze soaked in chlorhexidine	
Appropriate Wound Bed Preparation and Coverage of Exposed Tendons and underlying tissues	12	Meek micrografting technique for split-thickness skin graft (STSG) expansion, using the right thigh as the donor site	
	13	Engraftment of Meek micrografts	
	13–15	Re-epithelialization of each Meek graft occurred from its edges, gradually covering the wound bed.	
Impaired Wrist and Elbow Joints, and Inability to Extend First Finger Impaired Wrist and Elbow Joints, and Inability to Extend First Finger	15–41	Follow-up with a comprehensive physical therapy program	Elbow Joint: - Passive Flexion: 130° improved to 135° (6 months) - Passive Extension: 50° improved to 65° (6 months) Wrist Joint: - Passive Flexion: 25° improved to 40° (6 months) - Passive Extension: 30° improved to 40° (6 months) First finger: - No improvement due to severe damage to the extensor tendon.
	41–67	Follow-up with a comprehensive physical therapy program	Elbow Joint: - Passive Flexion: 130° improved to 140° (1 year) - Passive Extension: 50° improved to 80° (1 year) Wrist Joint: - Passive Flexion: 25° improved to 60° (1 year) - Passive Extension: 30° improved to 50° 1 year) First finger: - No improvement due to severe damage to the extensor tendon.

## Data Availability

The data presented in this study are available on request from the corresponding author.
